# Superior Heavy Metal Ion Adsorption Capacity in Aqueous Solution by High-Density Thiol-Functionalized Reduced Graphene Oxides

**DOI:** 10.3390/molecules28103998

**Published:** 2023-05-10

**Authors:** Ho-Geun Kim, Jong-Seong Bae, Injoo Hwang, Sung-Hoon Kim, Ki-Wan Jeon

**Affiliations:** 1 Department of Advanced Technology and Engineering, Graduate School, Silla University, Busan 46958, Republic of Korea; sksmsghrms123@naver.com; 2 Busan Center, Korea Basic Science Institute, Busan 46742, Republic of Korea; jsbae@kbsi.re.kr; 3 Department of Mechanical Engineering, Silla University, Busan 46958, Republic of Korea; hwanginjoo@silla.ac.kr

**Keywords:** heavy metal ion removal, thiol functionalization, graphene, adsorption, metal–sulfur bond dissociation energy

## Abstract

The preparation of mercapto-reduced graphene oxides (***m*-RGOs**) via a solvothermal reaction using P_4_S_10_ as a thionating agent has demonstrated their potential as an absorbent for scavenging heavy metal ions, particularly Pb^2+^, from aqueous solutions due to the presence of thiol (–SH) functional groups on their surface. The structural and elemental analysis of ***m*-RGOs** was conducted using a range of techniques, including X-ray diffraction (XRD), Raman spectroscopy, optical microscopy, scanning electron microscopy (SEM), transmission electron microscopy (TEM), scanning transmission electron microscopy equipped with energy-dispersive spectroscopy (STEM-EDS), and X-ray photoelectron spectroscopy (XPS). At pH 7 and 25 °C, the maximum adsorption capacity of Pb^2+^ ions on the surface of ***m*-RGOs** was determined to be approximately 858 mg/g. The heavy metal–S binding energies were used to determine the percent removal of the tested heavy metal ions, with Pb^2+^ exhibiting the highest percentage removal, followed by Hg^2+^ and Cd^2+^ ions having the lowest percent removal, and the binding energies observed were Pb–S at 346 kJ/mol, Hg–S at 217 kJ/mol, and Cd–S at 208 kJ/mol. The time-dependent removal study of Pb^2+^ ions also yielded promising results, with almost 98% of Pb^2+^ ions being removed within 30 min at pH 7 and 25 °C using a 1 ppm Pb^2+^ solution as the test solution. The findings of this study clearly demonstrate the potential and efficiency of thiol-functionalized carbonaceous material for the removal of environmentally harmful Pb^2+^ from groundwater.

## 1. Introduction

Water contamination by heavy metal ions, even at trace levels, has become a serious global environmental issue due to the toxicity and non-biodegradability of these ions, which have a tendency to accumulate in organs such as the heart, liver, and brain [1,2,3]. Therefore, the effective removal of hazardous heavy metals from the environment, especially from water and wastewater, has become essential for the welfare of living organisms [4]. Until now, several methods and techniques have conventionally been used in treating wastewater and removing hazardous heavy metals from water, such as physicochemical adsorption [5,6], chemical precipitation [7], membrane filtration [8,9], ion exchange [10], bio-removal [11], catalytic reduction [12], and electrocoagulation [13]. Among the aforementioned methods, adsorption is considered an attractive approach and is extensively employed in industry for its low cost, ease of operation, high removal efficiency, long-term stability, and because it does not introduce secondary pollution [3,6]. Several studies have resulted in the utilization of various adsorbents for removing heavy metals from solutions, primarily through physicochemical adsorption facilitated by their high surface area. These adsorbents include activated carbon [14,15], fly ash [16], sawdust [17], crab shell [18], coconut shell [19], sugarcane bagasse [20], zeolite [21], rice husk [22], and iron and manganese oxides [23]. However, these adsorbents show poor removal efficiencies for metal ions at low concentrations.

Compared with other adsorbents, graphene oxides (GOs) are considered the most promising adsorbent for removing heavy metals from water due to their large theoretical specific surface area and large number of various oxygen functional groups, including hydroxyl and carboxyl groups [6]. To further enhance their ability to adsorb heavy metal ions, GOs undergo surface modification with organic molecules [6], layered double hydroxides [24], and nanoparticles [25]. However, the adsorption capacity of such GO-based composites is still insufficient for practical use due to their lack of acidic oxygen functional groups. In order to significantly enhance the adsorption capacity of GOs, functional groups containing an acidic proton should be covalently bonded to the surface of GOs at a high density. Furthermore, functional groups with high affinity for heavy metal ions and easily deprotonated properties are more likely to attract heavy metal ions compared to oxygen functional groups. Taking the aforementioned into account, previous studies have demonstrated that the adsorption capacity of heavy metal ions in aqueous solutions can be improved through surface modification of GOs with organic molecules terminated with thiol groups [26,27].

In this study, we report for the first time the efficient utilization of –SH functionalized graphene, namely mercapto-reduced graphene oxides (***m*-RGOs**), for the scavenging of Pb^2+^ ions from water. We have succeeded in exploiting the high binding affinity of Pb^2+^ ions for the surface–SH groups of ***m*-RGOs** (binding energy of the Pb–S bond: 398 kJ·mol^−1^) for this purpose [28]. The crucial role of the surface–SH groups of ***m*-RGOs** in Pb^2+^ ion scavenging is explained in great detail in this report. The effectiveness of ***m*-RGOs** as efficient heavy metal ions that scavenge carbon nanomaterials has been studied in great detail by varying the concentration of Pb^2+^ ions in water. Furthermore, the effect of the pH of an aqueous solution has been investigated to determine the optimal pH range required for heavy metal removal. The contact time was varied in order to determine the time required to reach the maximum adsorption of Pb^2+^ ions on ***m*-RGOs**, i.e., equilibrium or saturation. The results showed that the highest adsorption capacity for Pb^2+^ ions (858.12 mg/g) was obtained using ***m*-RGOs** as a single-component system at pH 7 at 25 °C, which is 100 times higher than the highest adsorption capacity of reduced graphene oxides (RGOs) and 15 times higher than that of RGO aerogel, as reported in the literature [6,29,30,31,32].

## 2. Results and Discussion

### 2.1. Structural Characterization of **m-RGOs**

**Figure 1:**
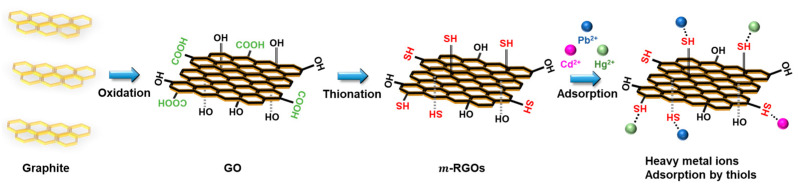
Illustration of the synthesis of ***m*-RGOs** and removal of heavy metal ions.

In Figure 1, the synthetic procedure for ***m*-RGOs** is shown, along with the process for removing heavy metal ions. The experimental details for both the oxidation of graphite and the thionation of GOs were addressed in our previous report [33].

**Figure 2:**
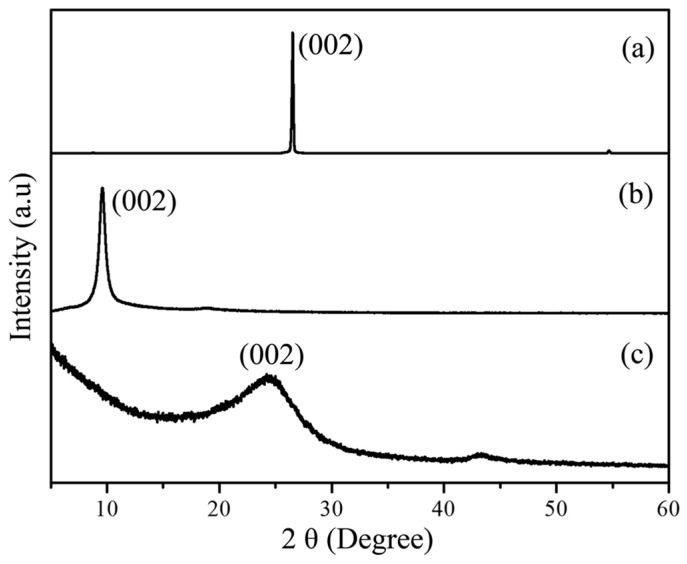
Powder X-ray diffraction of (**a**) graphite, (**b**) GOs, and (**c**) ***m*-RGOs**.

The XRD study (Figure 2) clearly indicates by comparison of the (002) diffraction peak position that graphite was fully oxidized and the GOs were reduced after the solvothermal reaction.

**Figure 3:**
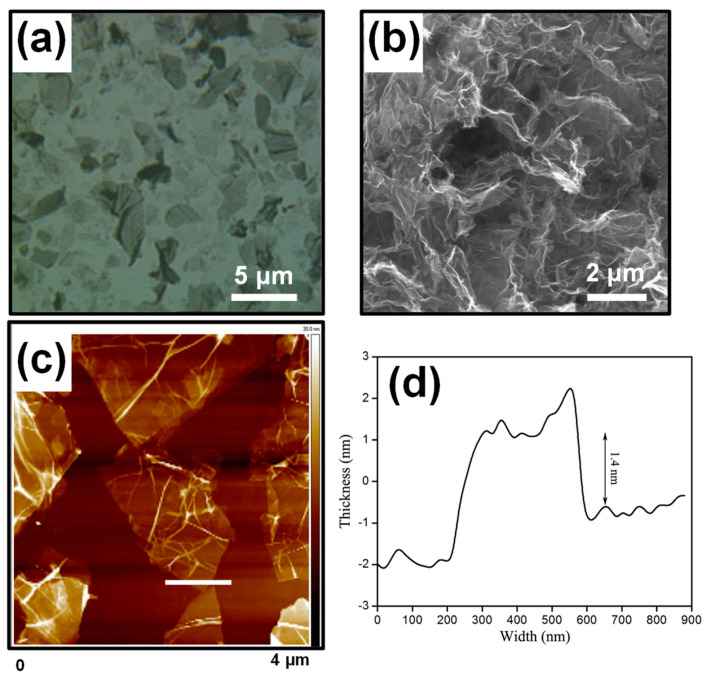
Structural images of ***m*-RGOs** using (**a**) optical microscopy, (**b**) SEM, (**c**) AFM, and (**d**) the height profile of the selected area in (**c**).

**Figure 4:**
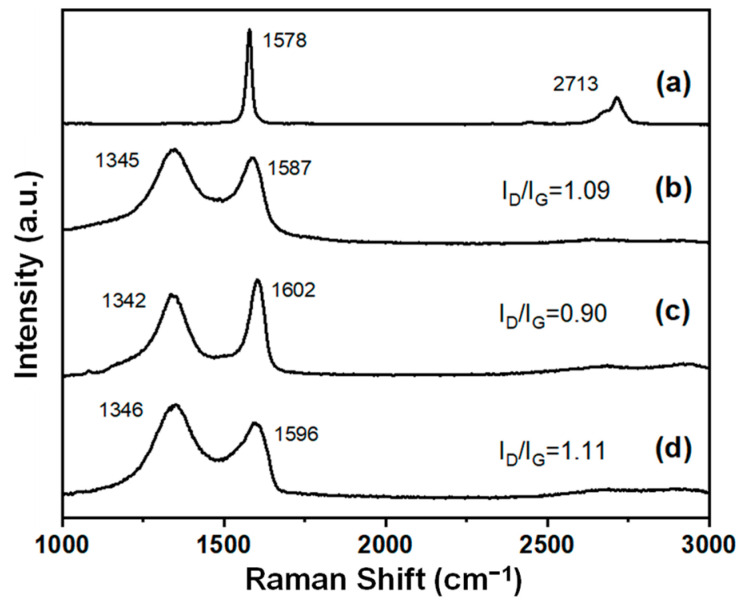
Raman spectra for (**a**) graphite flakes, (**b**) GOs, (**c**) RGOs, and (**d**) ***m*-RGOs**.

The weak intensity and broad width of the (002) peak shown at approximately 24 degrees (Figure 2c) were due to irregular sheet stacking along the c-axis. To carefully investigate the morphology of the ***m*-RGOs**, we carried out optical microscopy, SEM, and AFM (Figure 3). The typical sheet size of ***m*-RGO** sheets was estimated by optical microscopy to be approximately 3 μm (Figure 3a). The morphology of the ***m*-RGOs** as determined using SEM is shown in Figure 3b: it is flake-like with irregular edges and wrinkles. We used AFM to further examine the morphology and thickness of the ***m*-RGOs**. The AFM image and corresponding height profile of the ***m*-RGOs** are shown in Figure 3c,d, respectively. The ***m*-RGO** sheets were completely exfoliated after sonication for about 30 min in ethanol, as shown by AFM. The thickness of the ***m*-RGOs** is around 1.4 nm, which corresponds to the previously reported thickness of monolayer graphene. Additionally, an investigation of the Raman spectra of the graphite, GOs, RGOs, and ***m*-RGOs** showed changes in the intensity and position of the D and G bands, which indicates some degree of functionalization on the surface of the graphene sheets. The Raman spectrum of graphite shown in Figure 4a shows only G and 2D bands, which is typical. GOs (Figure 4b) show D and G bands at around 1350 and 1590 cm^−1^, respectively, indicating the presence of defects and disorder on the sheets. The thermal reduction process of GOs to produce RGOs was conducted under the same experimental conditions used for ***m*-RGOs** without P_4_S_10_. In the present study, the I_D_/I_G_ ratio of RGOs was determined to be 0.9, while the I_D_/I_G_ ratio of GOs was found to be 1.09. This observation indicates that RGOs have a lower degree of defects and disorder than GOs. The Raman spectrum of the ***m*-RGOs** shown in Figure 4d exhibits a high I_D_/I_G_ ratio of 1.11, implying that a comparable number of defects are present on the ***m*-RGOs** sheets after concomitant reduction and thionation, as on GOs. To achieve better insight into ***m*-RGOs**, it is necessary to examine the origin of their numerous defects. Therefore, an elemental analysis of ***m*-RGOs** was carried out using X-ray photoelectron spectroscopy (XPS).

### 2.2. Elemental Analysis of **m-RGOs**

**Figure 5:**
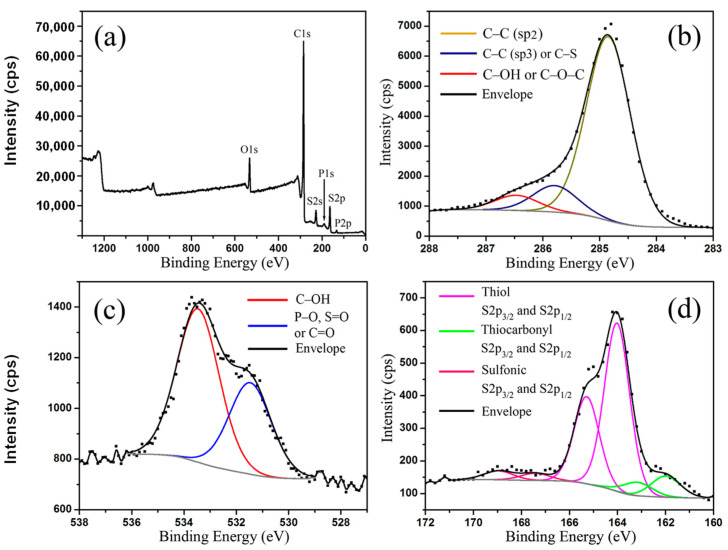
XPS spectrum of ***m*-RGOs**; (**a**) High resolution XPS spectra for (**b**) C1s, (**c**) O1s, and (**d**) S2p.

To more deeply investigate the numerous defects on the surface of the ***m*-RGOs**, XPS analysis was performed. The scan of the ***m*-RGOs** shown in Figure 5a indicates the existence of carbon, oxygen, sulfur, and phosphorous elements. The C:O:S and C:(O+S) atomic ratios for ***m*-RGOs** as calculated from the scan were 12.7:1:1.2 and 5.8:1, respectively. Furthermore, the C:O atomic ratio of the RGOs (Figure S1) was found to be 6.6:1, which supports their lower I_D_/I_G_ ratio than that of ***m*-RGOs**. The deconvoluted high-resolution XPS spectrum of the C1s shown in Figure 5b reveals a broad FWHM envelope, indicating the presence of several different types of carbon species on the surface, including oxygen and sulfur functional groups. The major component is sp^2^-hybridized carbon, which accounts for 75% of the total carbon species. The high-resolution XPS spectrum of O1s, deconvoluted and depicted in Figure 5c, shows that there are several types of oxygen species, which could be attributed to C–O, C=O, P–O, and/or S=O. Upon the deconvolution of the high-resolution O1s spectrum of ***m*-RGOs**, it was determined that the two major oxygen-containing functional groups present on the surface are hydroxyl (−OH) and carbonyl (−C=O), and they account for 60% and 40% of the atomic composition, respectively. The high-resolution S2p XPS (Figure 5d) clearly shows the presence of thiol (−SH), thiocarbonyl (C=S), and sulfonic acid (−SO_3_H) groups on the ***m*-RGO** surface, with SH as the major (83 atomic %) sulfur component.

### 2.3. Importance of –SH Groups in Pb^2+^ Ion Removal

**Figure 6:**
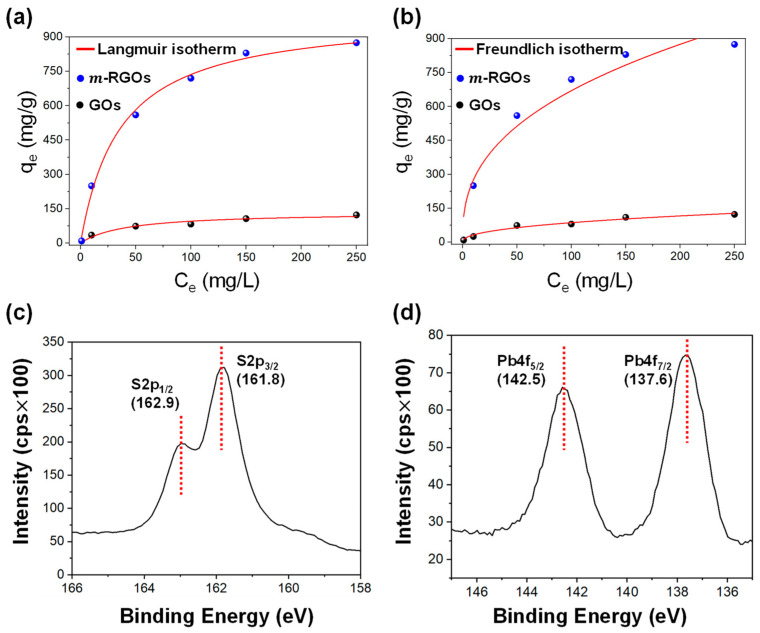
(**a**) Langmuir, (**b**) Freundlich isotherm model for Pb(II) adsorption on GOs and ***m*-RGOs**, and high resolution XPS of (**c**) S2p and (**d**) Pb4f for ***m*-RGOs** after Pb(II) adsorption.

The functional groups found on the surface of ***m*-RGOs**, especially –SH, were identified in our current research as playing a vital role in removing heavy metal ions such as Pb^2+^ from water. Determining the maximum uptake capacity is a crucial parameter in evaluating the performance of adsorbents. Thus, in our research, both the Langmuir and Freundlich isotherm models were employed to investigate the adsorption capacity of Pb^2+^ ions in water, as illustrated in Figure 6a,b. The adsorption isotherm for Pb^2+^ was investigated over a range of concentrations from 1 ppm to 250 ppm. Table 1 presents the results of the analysis using the Langmuir and Freundlich models. The results demonstrate that both models fit the experimental data well: the Langmuir and Freundlich models yielded correlation coefficients of 0.942 and 0.948, respectively. Notably, ***m*-RGOs** demonstrated a remarkably higher adsorption capacity for Pb^2+^ ions than did GOs, with maximum values of 858.12 mg/g and 121.12 mg/g, respectively. Although both GOs and ***m*-RGOs** displayed similar defects and levels of surface disorder (as shown in Figure 4) as well as surface area (Figure S2, GO: 138 m^2^/g and ***m*-RGOs**: 124 m^2^/g), our research indicates that ***m*-RGOs** exhibit a significantly higher adsorption capacity for Pb^2+^ ions in water, which can be attributed to their –SH functional groups.

A previous study suggested that the primary groups responsible for removing Pb^2+^ ions from water are the –COOH groups of GOs [34]. The low density of –COOH groups in GOs prepared in this study (3.6 atomic %) could result in a lower adsorption capacity. The surface of ***m*-RGOs** is enriched with –SH functional groups, which can significantly enhance the ***m*-RGO** adsorption capacity for Pb^2+^ ions. The maximum adsorption capacity achieved in this study with the use of ***m*-RGOs** as the adsorbent (858.12 mg/g) was significantly higher than the values that have been reported in the literature for other conventional adsorbents, such as activated carbon fibers (52.7 mg/g) [29], graphene oxide-ethylenediaminetetraacetic acid (GO-EDTA) (479 mg/g) [6], carbon nanotubes (15.6 mg/g) [31], multiwalled carbon nanotubes (3.0 mg/g) [32], and GO-PVA-SH composite (218 mg/g) [27]. This result highlights the superior efficiency of our new thiol-functionalized graphitic material for heavy metal removal relative to the aforementioned adsorbents.

Furthermore, the strong affinity of –SH functional groups for Pb^2+^ ions (with a binding energy of 398 kJ/mol for the Pb–S bond) may further enhance adsorption capacity. After the adsorption of Pb^2+^ ions onto ***m*-RGOs**, the binding energies of S2p_3/2_ and Pb4f_7/2_ were determined to be 161.8 eV and 137.6 eV, respectively, which is indicative of the formation of PbS, as shown in Figure 6c,d.

### 2.4. Effect of pH on the Percent Removal of Pb^2+^ Ions Using **m-RGOs**

The pH of the aqueous solution plays a critical role in the adsorption of Pb^2+^ onto ***m*-RGOs**. We observed that an increase in pH results in an enhancement of the adsorption of metallic species onto most adsorbents, as reported in the literature [6,35]. The effect of pH on the percent removal of Pb^2+^ by ***m*-RGOs** was investigated. As shown in Figure S1, the percent removal of Pb^2+^ from water is around 10–20% when the pH of the solution lies within the pH range of 2–3. However, there is a significant increase in the removal of Pb^2+^ to approximately 50% when the pH is increased to 5. At a highly acidic pH (pH = 2–3), the –SH groups that are present on the surface of ***m*-RGOs** are only slightly deprotonated in the aqueous solution, which results in relatively lower adsorption of Pb^2+^ on ***m*-RGOs**.

On the other hand, when the pH is 6, the adsorption of Pb^2+^ onto ***m*-RGOs** significantly increases. At this pH, some of the –SH groups are deprotonated, which provides better electrostatic interactions between the deprotonated –SH groups and Pb^2+^. This interaction makes the adsorption of Pb^2+^ onto ***m*-RGOs** more favorable. At a neutral pH (pH = 7), the percent removal of Pb^2+^ is almost 100%. We found that at high pH values, e.g., pH = 9–10, ***m*-RGOs** exhibited complete removal of Pb^2+^ from the water. Furthermore, some precipitation of Pb^2+^ ions as Pb(OH)_2_ was also observed within this basic pH range [6]. Therefore, the removal of Pb^2+^ at highly basic pH ranges cannot be completely attributed to the presence of ***m*-RGOs** alone but is also due to the precipitation of the heavy metal, whereas the high Pb^2+^ adsorption onto ***m*-RGOs** at pH 7 can mostly be attributed to the higher affinity of –SH groups for Pb^2+^, along with the Coulombic attraction force between the deprotonated –SH groups and Pb^2+^, as mentioned earlier.

### 2.5. Effect of Contact Time on the Percentage Removal of Pb^2+^ Using m-RGOs

**Figure 7:**
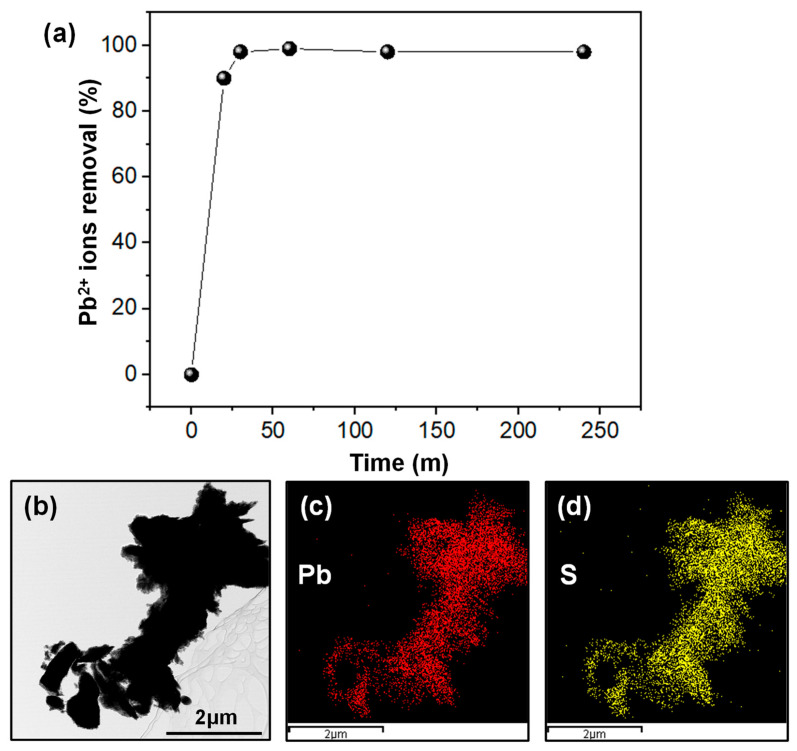
(**a**) Effect of contact time on the percent removal of Pb^2+^ by ***m-RGOs*** and (**b**–**d**) TEM images of ***m-RGOs*** after Pb^2+^ adsorption and corresponding elemental mapping images.

The impact of contact time on the percentage removal of Pb^2+^ by ***m*-RGOs** was investigated, and the findings are presented in Figure 7a. To carry out this study, a 1 ppm Pb^2+^ solution was allowed to interact with the adsorbent for durations ranging from 10 to 240 min at 25 °C and a pH of 7. Our results demonstrate that nearly 99% of Pb^2+^ can be removed by ***m*-RGOs** within 30 min under these experimental conditions. This suggests that adsorption equilibrium is rapidly achieved, which indicates efficient adsorption of Pb^2+^ by the –SH functional groups situated on the surface of ***m*-RGOs**. These outcomes also indicate that the time required for ***m*-RGOs** to attain adsorption equilibrium is lower than that of conventional adsorbents such as activated carbon (approximately 4 h) [30] and multiwalled carbon nanotubes (8 h) [32]. The short equilibrium adsorption rate for ***m*-RGOs** makes this adsorbent a promising and efficient candidate for heavy metal removal from wastewater. In addition, after adsorption, TEM was carried out and its corresponding elemental mapping images were obtained, which indicated that Pb^2+^ ions were captured by thiol functional groups (Figure 7b–d). Furthermore, a comparative analysis of the percent removal of various heavy metal ions, including Pb^2+^, Hg^2+^, and Cd^2+^, was conducted (see Figure S3). The results indicated that the order of percent removal for these heavy metal ions was as follows: Pb^2+^ exhibited the highest percent removal, followed by Hg^2+^, and then Cd^2+^, which had the lowest percent removal. Interestingly, it was observed that the binding energy between the sulfur and each heavy metal ion followed the same order as the percent removal of these ions (Pb–S: 346 kJ/mol, Hg–S: 217 kJ/mol, Cd–S: 208 kJ/mol).

## 3. Materials and Methods

### 3.1. Synthesis of **m-RGOs**

The graphene oxides (GOs) utilized in this study were synthesized via the modified Hummers method, as previously described in our earlier publication [34]. As-prepared GO in water was mixed with a 1 M NaOH solution to obtain precipitated GO, and subsequently, the pH of the precipitated GO was adjusted to around 9 by repeated washing with deionized water by centrifugation. The solvent exchange of the wet GO gel containing water was performed with pyridine (Alfa-aesar, Haverhill, MA, USA, ACS reagent, ≥99.0%) by repeated ultrasonication and centrifugation. The washed GO gel with pyridine was homogenized for 5 min to obtain a homogeneous GO suspension for further thiolation reactions. For the synthesis of ***m*-RGOs**, phosphorus decasulfide (P_4_S_10_, Sigma-Aldrich, St. Louis, MO, USA, 99%) has been used as a source of sulfur. The ***m*-RGOs** samples were synthesized by using a 10% excess with respect to a stoichiometric amount of P_4_S_10_. In a typical solvothermal reaction, 110 mg of P_4_S_10_ was slowly added to 13 mL of GO suspension (~7.7 mg/mL) in a Teflon-lined autoclave of 23 mL capacity. It was then placed in a pre-heated oven at 180 °C for 12 h. After the reaction, the solid product was collected via vacuum filtration and washed with deionized water and ethanol several times to remove all the unreacted precursors and by-products. The ***m*-RGOs** in deionized water were subsequently freeze-dried to avoid re-stacking the graphene sheets, and then they were undergone for further characterization and uses.

### 3.2. Characterization of **m-RGOs**

X-ray photoelectron spectroscopic (XPS) measurements were carried out using a VG-220IXL spectrometer with monochromated Al Kα radiation (1486.6 eV, line width 0.8 eV). The pressure in the analyzing chamber was kept at 10^−9^ torr while recording the spectra. The spectrometer has an energy resolution of 0.4 eV. All binding energies were corrected with reference to C(1s) at 284.6 eV. The Shirley background was used for deconvolution. Surface topography images were obtained using an atomic force microscope (Pico-Plus AFM, Molecular imaging, Agilent Technologies, Santa Clara, CA, USA). All AFM studies were performed in air in tapping mode with SCANASYST-AIR tips (Bruker, Billerica, MA, USA). The images were collected at a scan rate of 1.0 Hz in air. Scanning transmission electron microscopy (STEM) images and elemental mapping images were acquired using a JEOL 2010F, Akishima, Tokyo, Japan (200 kV) TEM/STEM equipped with a Schottky-type field emission gun and an EDAX thin window X-ray energy dispersive spectrometer (EDS) detector. The Raman data were collected using a custom-built Raman spectrometer in 180° geometry. The sample was excited using a 0.75 mW Compass 532 nm laser. Nitrogen sorption isotherms were collected on a Micromeritics ASAP 2020 volumetric adsorption analyzer at 77 K. Prior to testing, all samples underwent a 12 h outgassing process at room temperature under vacuum until the residual pressure reached ≤10 μmHg. The specific surface areas were then determined using the Brunauer-Emmett-Teller (BET) equation within the relative pressure range of 0.06 to 0.2.

### 3.3. Adsorption Experiments

To investigate the sorption ability and mechanism of ***m*-RGOs** as adsorbents, batch adsorption experiments were conducted using heavy metal standard solutions with an initial concentration range of 1 to 250 ppm, which were prepared by diluting a stock solution of 1000 ppm. The pH effect was studied by adjusting the pH of the aqueous solution using 0.01 M NaOH and HCl. The batch adsorption experiments were conducted by dispersing 1 mg of ***m*-RGOs** in 10 mL of nano-pure water containing different concentrations (1–250 ppm) of Pb^2+^ solutions (pH = 7) with an adsorbent dose of 0.1 mg/mL at 25 °C. The mixture was sonicated for 30 min to ensure a homogeneous dispersion of ***m*-RGOs**, followed by a batch adsorption process in a mechanical shaker (400 rpm) for 4 h. After filtration through a 0.22 µm pore size membrane, the concentration of Pb^2+^ in the filtrates was measured by an inductively coupled plasma-optical emission spectrometer (ICP-OES). The amount of Pb^2+^ adsorbed was determined by calculating the difference between the initial and equilibrium concentrations of Pb^2+^. We note that batch adsorption experiments with GOs were conducted under the same experimental conditions used for ***m*-RGOs**. The percentage removal was calculated as the ratio of the adsorbed amount to the initial concentration, multiplied by 100. The experiments were performed in triplicate to ensure reproducibility.

The adsorption capacity ($q_{e}$) was determined from the following equation:

(1) $$q_{e}=\frac{\left(C_{i}-C_{e}\right)\times V}{m}$$

where $C_{i}$ and $C_{e}$ are the initial and equilibrium concentration (ppm) of heavy metal ions, respectively. V is the volume (mL) of heavy metal solution and m is the mass (mg) of ***m*-RGOs**.

The adsorption parameters for Langmuir fit were estimated by Equation (2):

(2) $$q_{e}=\frac{q_{max}K_{L}C_{e}}{1+K_{L}C_{e}}$$

where $q_{e}$ is the adsorption amount of heavy metal ions onto ***m*-RGOs** adsorbent (mg/g) at equilibrium, $q_{max}$ is the maximum uptake capacity of heavy metal ions on adsorbent (mg/g), $C_{e}$ is the equilibrium concentration of heavy metals, and *K_L_* is the Langmuir adsorption constant, which is related to adsorption energy.

The Freundlich model is shown in Equation (3):

(3) $$q_{e}=K_{F}C_{e}^{1/n}$$

where $q_{e}$ is the adsorption amount of heavy metal ions onto ***m*-RGOs** adsorbent (mg/g) at equilibrium, $C_{e}$ is the equilibrium concentration of heavy metals ions, and $K_{F}$ and n are Freundlich constants that are related to adsorption energy and adsorption intensity, respectively.

## 4. Conclusions

The present study provides a detailed investigation of the adsorption of Pb^2+^ onto ***m*-RGOs** and reveals that the material exhibits promising adsorption capacity in aqueous solution due to the presence of –SH groups on its surface. Additionally, the results demonstrate an increase in the percentage of Pb^2+^ ions removed with decreasing initial concentrations of Pb^2+^ ions and indicate that the chemical interaction between the –SH functional groups on the surface of ***m*-RGOs** and the Pb^2+^ ions is highly pH-dependent. The highest adsorption capacity of Pb^2+^ ions by ***m*-RGOs** was found to be 858 mg/g at pH 7 and at 25 °C. The heavy metal–S binding energies were used to determine the percent removal of the tested heavy metal ions, with Pb^2+^ exhibiting the highest percentage removal, followed by Hg^2+^ and Cd^2+^ ions, which had the lowest percent removal, and the binding energies observed were Pb–S at 346 kJ/mol, Hg–S at 217 kJ/mol, and Cd–S at 208 kJ/mol. Furthermore, promising results were obtained from the kinetic studies of the percentage of Pb^2+^ ions removed at pH 7 and at 25 °C. Taken together, these findings suggest that ***m*-RGOs** have significant potential as an excellent adsorbent for the effective removal of heavy metal ions, including Pb^2+^, from both groundwater and wastewater.

## Figures and Tables

**Table 1:** Parameters of Langmuir and Freundlich models for the adsorption of Pb^2+^ onto **m-RGOs** and GOs.

Adsorbents		Langmuir		Freundlich		
	K_L_ (L/mg)	q_max_ (mg/g)	R^2^	K_F_ (mg/g) (mg/L)^1/n^	n	R^2^
***m*** **-RGOs**	0.028	998	0.995	113	2.59	0.958
**GO**	0.024	135	0.957	11	2.13	0.966

## Data Availability

The data that support the findings of this study are available from the corresponding authors upon reasonable request.

## Supplementary Materials

The following supporting information can be downloaded at: https://www.mdpi.com/article/10.3390/molecules28103998/s1, Figure S1: Effect of pH on the percent removal of Pb^2+^ by ***m*-RGOs**; Figure S2: BET N_2_ adsorption-desorption isotherms for (a) GOs and (b) m-RGOs; Figure S3: Percent removal of heavy metal ions by ***m*-RGOs**.

## Sample Availability

Samples of the compounds are available from the authors.
