# Supplementary material for: Superior Heavy Metal Ion Adsorption Capacity in Aqueous Solution by High-Density Thiol-Functionalized Reduced Graphene Oxides
Source: Molecules. 2023 May 10;28(10):3998. doi: 10.3390/molecules28103998 (PMC10220523; doi:10.3390/molecules28103998)
Supplement: Supplementary file 1 [file molecules-28-03998-s001.zip › molecules-2373158-supplementary.pdf]

Supporting Information

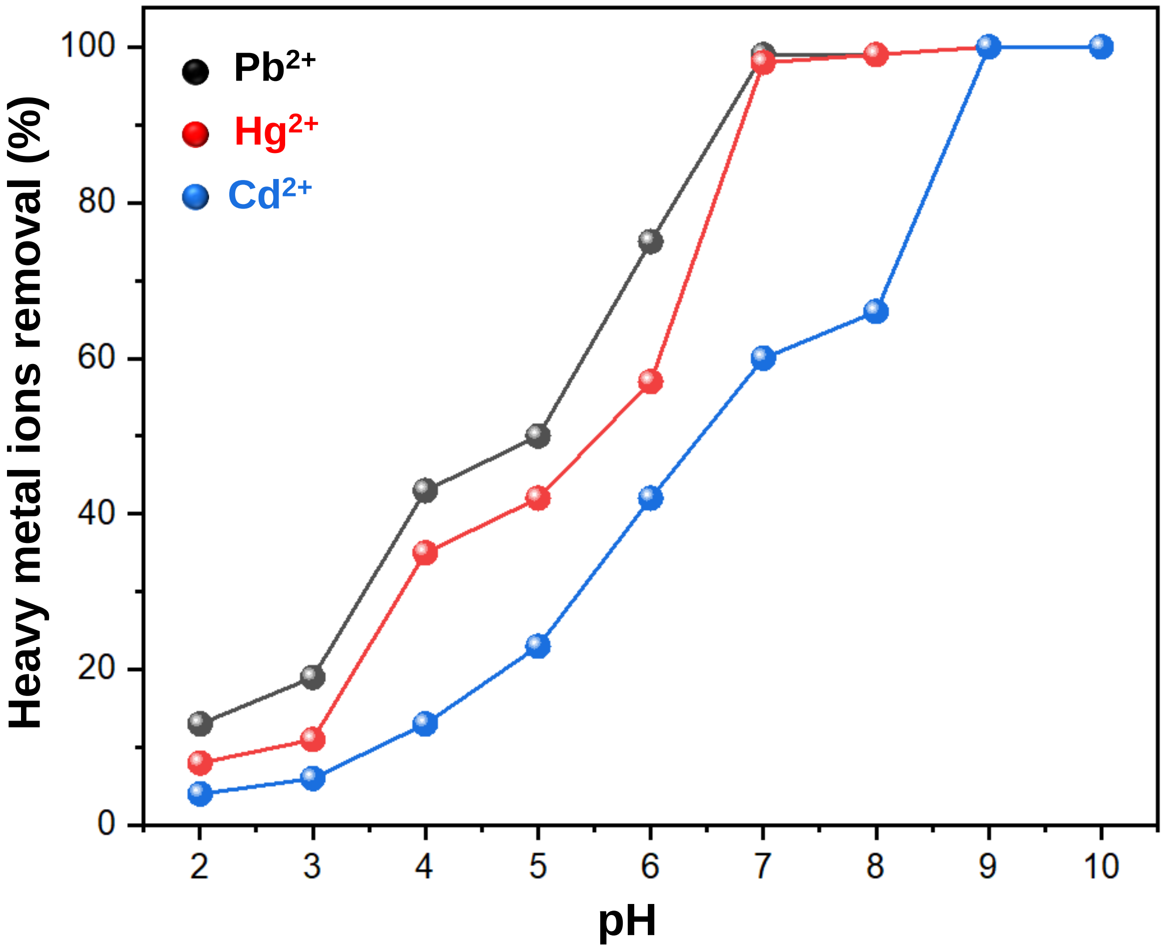

Figure S1. Effect of pH on the percent removal of Pb^{2+} by ***m*-RGOs**.

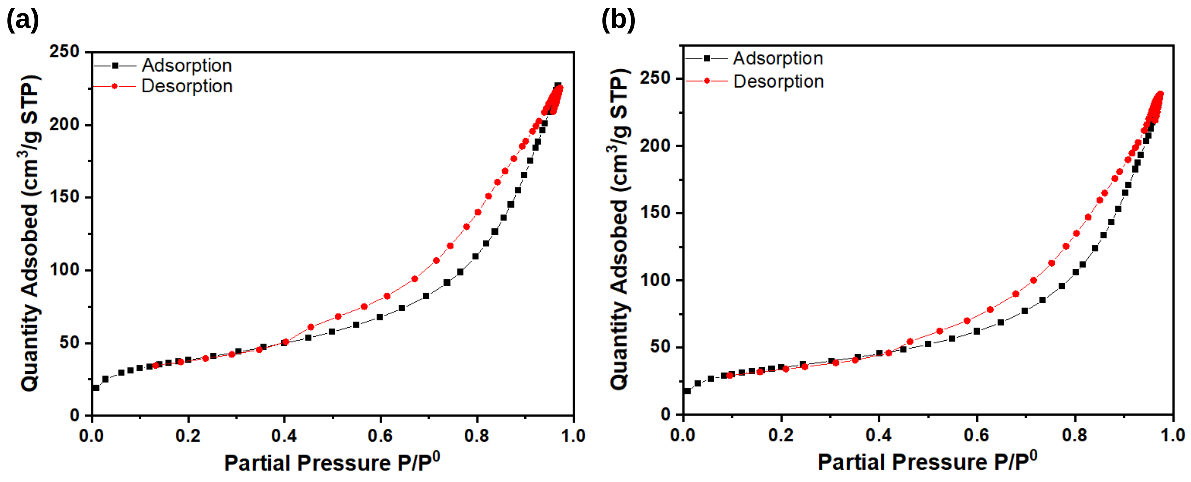

Figure S2. BET N_2 adsorption-desorption isotherms for (a) GOs and (b) ***m*-RGOs**.

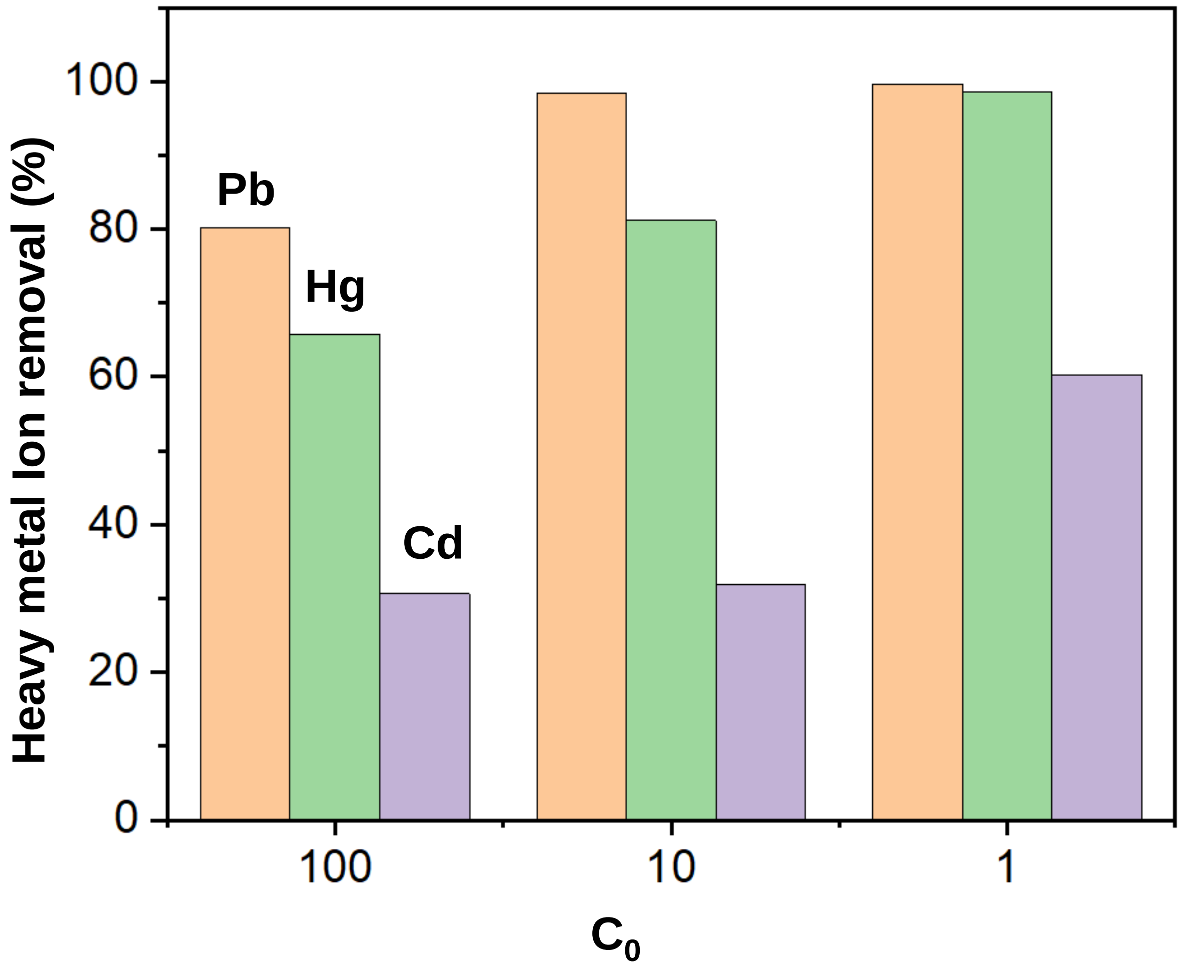

Figure S3. Percent removal of heavy metal ions by ***m*-RGOs**.
